# Association of wild bird densities around poultry farms with the risk of highly pathogenic avian influenza virus subtype H5N8 outbreaks in the Netherlands, 2016

**DOI:** 10.1111/tbed.13595

**Published:** 2020-05-18

**Authors:** Francisca C. Velkers, Thijs T. M. Manders, Johannes C. M. Vernooij, Julia Stahl, Roy Slaterus, J. Arjan Stegeman

**Affiliations:** ^1^ Department of Farm Animal Health Faculty of Veterinary Medicine Utrecht University Utrecht The Netherlands; ^2^ Sovon Dutch Center for Field Ornithology Nijmegen The Netherlands

**Keywords:** disease outbreaks, H5N8 subtype, Influenza A Virus, population density, poultry, wild birds

## Abstract

Highly pathogenic (HP) avian influenza viruses (AIV) can spread globally through migratory birds and cause massive outbreaks in commercial poultry. AIV outbreaks have been associated with proximity to waterbodies, presence of waterfowl or wild bird cases near poultry farms. In this study, we compared densities of selected HPAI high‐risk wild bird species around 7 locations (H farms) infected with HPAIV H5N8 in the Netherlands in 2016–2017 to densities around 21 non‐infected reference farms. Nine reference farms were in low‐lying water‐rich areas (R‐W) and 12 in higher non‐water‐rich areas (R‐NW). Average monthly numbers/km^2^ of Eurasian wigeons, tufted ducks, *Anatidae* (ducks, geese and swans) and *Laridae* (gulls) were calculated between September and April in rings of 0–1, 1–3, 3–6 and 6–10 km around the farms. Linear mixed model analyses showed generally higher bird densities for H and R‐W compared to R‐NW farms between October and March. This was most striking for Eurasian wigeons, with in peak month December 105 (95% CI:17–642) and 40 (7–214) times higher densities around H and R‐W farms, respectively, compared to R‐NW farms. Increased densities around H farms for Eurasian wigeons and *Anatidae* were more pronounced for distances up to 10 km compared to 0–1 km that mostly consists of the farm yard, which is an unattractive habitat for waterfowl. This distance effect was not observed in gulls, nor in tufted ducks that live on large open waterbodies which are unlikely to be within 0–1 km of farms. This study provides insights into spatio‐temporal density dynamics of HPAI high‐risk birds around farms and their associations with poultry outbreaks. The outcomes indicate that knowledge of environmental and ecological drivers for wild bird presence and abundance may facilitate identification of priority areas for surveillance and biosecurity measures and decisions on establishments of poultry farms to reduce risk of HPAI outbreaks.

## 1 INTRODUCTION

**FIGURE 1:**
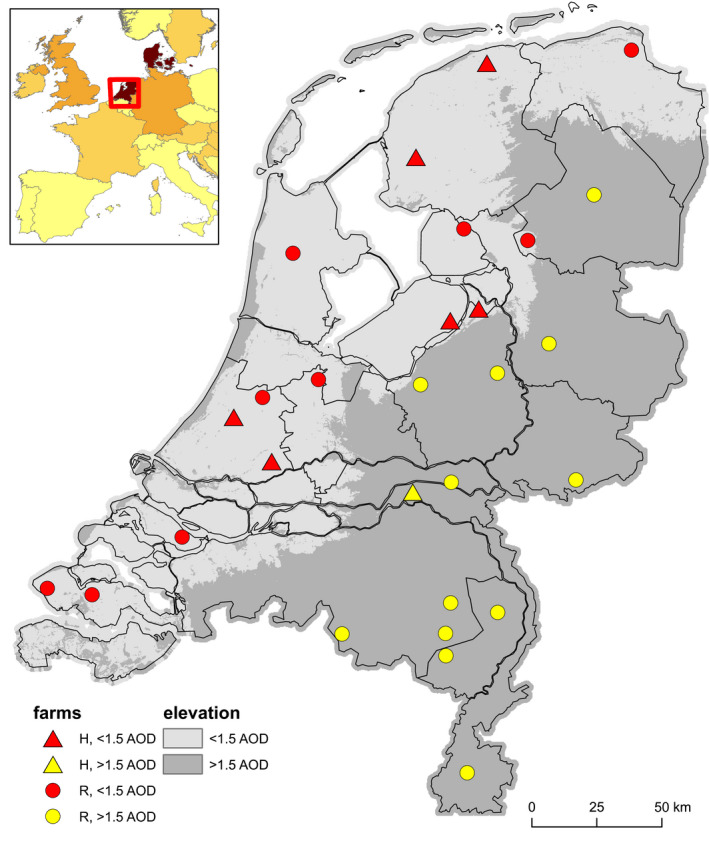
Location of hotspot and reference farms in relation to land elevation levels. Box in the left top shows an overlay of Europe with darker coloured countries indicating a larger wetland ratio, based on the Ramsar convention of wetlands. Red square includes the Netherlands. Main map shows the Netherlands, with grey shading indicating land elevation levels, based on the Amsterdam Ordnance Datum (AOD). Triangles indicate HPAI H5N8 outbreaks (hotspot, H locations) and circles reference farms. Red triangles or circles indicate locations in low‐lying parts of the Netherlands with AOD <1.5 (light grey), which are generally considered water‐rich. Yellow triangle and circles indicate locations in an area with AOD >1.5 (dark grey). The bird census schemes used to determine bird densities are visualized in the same map in Figure S1

Since 2014, highly pathogenic avian influenza A viruses (HPAIV) of clade 2.3.4.4 have spread globally causing massive outbreaks in commercial poultry (DeJesus et al., 2016; USDA‐APHIS, 2015). The movements of migratory birds have been shown to play and important role in inter‐continental and intracontinental dissemination of these HPAIVs (Lee, Bertran, Kwon, & Swayne, 2017; Lee et al., 2015; Mine et al., 2019). In the Netherlands, HPAIV H5N8 occurred from November 2014 onwards in commercial poultry holdings in water‐rich areas (Beerens et al., 2017). No increased wild bird mortality was observed, but phylogenetically related virus was found in faeces of live Eurasian wigeons (*Anas penelope)* in November 2014 (Poen et al., 2016; Verhagen et al., 2015). A novel reassortant HPAIV H5N8 emerged in 2016 in Asia, Europe, Africa and the Middle East causing massive wild bird die‐offs (FAO, 2018; Lee et al., 2017). In the Netherlands, the first wave of wild bird deaths due to H5N8 started on 8 November 2016 at large lakes in the middle of the country (Kleyheeg et al., 2017). Tufted ducks (*Aythya fuligula*) dominated the first wave of mortality; it was estimated that 85% of about 5,300 carcasses between 8 and 18 November consisted of tufted ducks, and the rest consisted of other species of the family of *Anatidae*, including ducks, swans and geese, and coots, grebes and gulls (Kleyheeg et al., 2017). From 21 November onwards, die‐offs mostly occurred in water‐rich agricultural areas and were dominated by Eurasian wigeons. The highest peak mortality of Eurasian wigeons occurred in the first half of December. Other species found in the second wave included other *Anatidae*, and scavengers such as gulls, raptors and corvids (Kleyheeg et al., 2017). The last reported wild bird case was a mallard (*Anas platyrhynchos*) on 15 March 2017 (OIE, 2017). Despite immediate indoor confinement and increased biosecurity, outbreaks in commercial poultry occurred between 25 November and 24 December 2016 in four fattening duck, one broiler breeder and three laying hen farms and a backyard poultry and waterfowl trading company. Ten outbreaks were reported in captive birds, that is backyard chickens and waterfowl, until the end of March 2017. Phylogenetic analyses indicated separate introductions from wild birds on most holdings (Beerens et al., 2017). Most commercial holdings were located in water‐rich areas, with lakes, sea shores, river floodplains, waterways, streams and ditches, and large (water) bird populations nearby (Figure 1).

Several studies have shown associations between proximity to waterbodies and presence of waterfowl or wild bird cases near farms and HPAI outbreaks (Bouwstra et al., 2017; Gilbert & Pfeiffer, 2012; Gonzales, Stegeman, Koch, de Wit, & Elbers, 2013; Mulatti et al., 2018; Napp, Majó, Sánchez‐Gónzalez, & Vergara‐Alert, 2018; Shimizu, Hayama, Yamamoto, Murai, & Tsutsui, 2018; Si, de Boer, & Gong, 2013; Ward, Maftei, Apostu, & Suru, 2008, 2009). Evidence from these studies suggests that wild bird density around farms could serve as a measure of exposure to HPAIV and, consequently, may be used to identify farms at high risk of virus introduction. For example, Galletti et al. (2018) showed that the density of AIV‐infected dabbling ducks (DID) had a high probability to be a risk factor for AIV introduction in poultry flocks and that 74% of primary (HP and LP) AI outbreaks over 17 years in Italy occurred in areas with a high DID. This suggests that the density of potentially AIV‐infected wild birds can be used to help identify risk areas for virus introduction.

The situation for the Netherlands is rather different from the countries where associations between wild birds and AIV were previously studied. A large part of the country consists of low‐lying water‐rich areas; however, poultry farms are mostly located in non‐wetland areas. As the 2016–2017 HPAI H5N8 epidemic in the Netherlands was characterized by mostly primary outbreaks on poultry farms, mainly occurring in wetland areas, the focus of this study was especially to identify differences in bird densities between outbreak farms and other farms located in wetland or non‐wetland areas. With only a limited number of HPAI H5N8 outbreaks in 2016–2017, a large‐scale analysis was not possible, so we used linear regression models to compare the average monthly density of specific wild bird species in autumn and winter months around seven HPAI H5N8 outbreak locations in 2016 in the Netherlands and 21 reference farms.

We specifically focused on four groups of wild birds, including the Eurasian wigeons and tufted ducks, *Anatidae* (including geese and swans, in addition to ducks) and gulls. In these bird groups, H5N8 virus was detected, but die‐offs occurred at different times during the epidemic, suggesting that they may have played different roles in the complex transmission dynamics at the wildlife/domestic interface and may include birds that introduced the virus to the Netherlands through migration, or acted like spreaders, maintenance or bridge hosts of the virus (Alarcon et al., 2018; Caron, Grosbois, Etter, Gaidet, & de Garine‐Wichatitsky, 2014; Lisovski et al., 2018). This approach helped to identify in which months and parts of the Netherlands specific birds were present in high densities. This study provides relevant insights into the spatio‐temporal density dynamics of HPAI high‐risk birds and their potential association with outbreaks in poultry.

## 2 METHODS

### 2.1 Study population

Seven infected commercial holdings (three duck farms located on the same road within 2.7 km were considered as one) were defined as outbreak (‘hotspot’, H) locations (OIE, 2017). We randomly selected uninfected poultry farms from the Netherlands Food and Consumer Product Safety Authority (NVWA) database. With the constraints of the limited number of H locations, we chose to use a stratified sample of 21 uninfected farms (‘reference’, R) for comparisons. Stratification was based on coverage of all provinces and various poultry types. Broiler farms were excluded, as HPAI (H5N8) outbreaks are rare in broilers (EFSA AHAW Panel, 2017; Napp et al., 2018). This may be related to genetically reduced susceptibility of broilers compared to layers, ducks or turkeys, in combination with factors related to the production system, such as fewer outside‐to‐on‐farm exposure to contacts, or enhanced biosecurity (Bertran et al., 2016, 2018). The final selection of 21 R farms included layer, broiler (grand)parent, layer parent, rearing hen, broiler breeder rearing, fattening duck and meat turkey farms (Table S1). For all farms, a geodetic reference frame for land height, the Amsterdam Ordnance Datum (AOD, also used as a reference for the European Vertical Reference System), was determined using Geoweb NAPinfo (Rijkswaterstaat, the Netherlands). Farms with AOD level below 1.5, with reference level 0 corresponding approximately to sea level, were considered to be located in low‐lying parts of the Netherlands which are generally water‐rich and attractive for waterfowl. Nine R farms were located in low‐lying water‐rich areas (R‐W: AOD −1.9; +1.5). Twelve R farms were located in higher non‐water‐rich areas (R‐NW: AOD +4.8; +133.7). One of the seven H farms was not located in a low part of the Netherlands (AOD +4.6) but was located near many small waterways (Figure 1; Table S1).

### 2.2 Wild bird data

The bird groups Eurasian wigeons, tufted ducks, *Anatidae* and *Laridae* (Table S2) were chosen to represent ‘high‐risk’ birds, as more than 91% of the Dutch H5N8 wild bird cases in 2016–2017 were confirmed in ducks, geese or swans (family of *Anatidae*) and 7% in gulls (family of *Laridae*)*.* Eurasian wigeons and tufted ducks were affected most often (Kleyheeg et al., 2017; OIE, 2017).

For these bird groups, data from standardized wild bird counts, from 2011/2012 to 2015/2016, collected by means of systematic regular bird census schemes, and waterfowl and midwinter counts of Sovon (Nijmegen, the Netherlands) were used. These data were not collected for the purpose of this study, but are part of national census schemes, which are in place to meet requirements of the EU Habitat and Bird Directive (EU, 1992) to assess bird population numbers, distribution and their fluctuation over years (population trends). The Dutch Network for Ecological Monitoring (NEM) contains monitoring schemes for different species groups, including the Dutch Waterbird Monitoring Scheme (DWMS). These schemes follow a highly standardized (inter)nationally approved protocol, validated by independent bodies, that is for the Netherlands by the national statistical office (Statistics Netherlands, CBS). In the DWMS, migrating and overwintering waterbirds are counted monthly between September and April on important waterbodies nationwide throughout the year (van Roomen, Koffijberg, Noordhuis, & Soldaat, 2006). The scheme grants high levels of standardization of data collection as well as intense coverage in space and time. Although the whole of the Netherlands is divided into a fine grid of hundreds of counting areas, not all of these areas can actually be counted. Bird counts are available for most areas from September to April. The number of areas that are counted, that is the counting coverage, is highest every year in January during the international midwinter count. A complete overview of the census schemes for the Netherlands, and counting coverage, can be found in Figure S1.

When there are incidentally missing data points in either space or time (Soldaat, Visser, van Roomen, & van Strien, 2007), for instance when a counter is unavailable in a certain month, the data in the monitoring scheme are completed by means of imputation. This is a statistical technique developed by the national statistical office (Statistics Netherlands, CBS) that uses average numbers from other months in the same area, bird counts in the same month of previous years and counts in comparable (counted) areas. Imputation is part of the standard validated trend analysis procedure of the DWMS (Soldaat et al., 2007).

In addition, when in some areas in the Netherlands no counts or very few counts are available, long‐term data of similar sites, months or years are used to extrapolate these missing values, which is an internationally approved technique within wildlife census schemes (Méndez et al., 2015; Musgrove et al., 2011). With this technique, population densities are estimated based on a predictive model that uses cumulative long‐term information and environmental stratification based on different variables of the habitat for that specific geographical area, as described by Méndez et al. (2015). In our study, extrapolation was mostly done for non‐water‐rich areas with low counting coverage.

Around each of the H and R locations, independent buffer rings with a radius of 0–1, 1–3, 3–6 and 6–10 km, representing a surface of 3.14, 25.13, 84.82 and 201.06 km^2^, respectively, were defined. For each of the buffer rings around these locations, the average bird counts/km^2^ were obtained from the DWMS census data for each month from September to April. For the purpose of this study, we used the average bird densities calculated over a period of 5 years (the winter periods of 2011/2012 to 2015/2016) to obtain a robust dataset. As fluctuations in counts between years, due to adverse weather conditions or other disturbances on counting days are inevitable, using average numbers of several years in analyses is an accepted method for long‐term monitoring to buffer fluctuations between years due to extreme effects of missing values or accidental ‘zero’ values (Musgrove et al., 2011, 2013). As a buffer ring transects multiple counting areas, the degree of overlap between the buffer ring and the underlying counting areas was determined. The average bird densities within the buffer ring were defined by the relative contribution of these areas to the buffer ring. For this, we assumed that birds have an even distribution in space within counting areas, that is that (a) bird density was equal within a counting area and (b) that the spatial share of different counting areas represents the share of birds from that particular area within the buffer ring. The density of birds per km^2^, and not the absolute standardized bird counts, was used for further analyses to exclude effects due to differences in ring surfaces. All bird densities per km^2^ were log10‐transformed to normalize the data.

### 2.3 Data analysis

Linear mixed model analyses were done with the lme function from the nlme package (Pinheiro, Bates, DebRoy, & Sarkar, 2019) in R version 3.3.0 (R Core Team, 2017). For each of the four bird groups, that is the Eurasian wigeon, tufted duck, *Anatidae* and *Laridae,* separate models were built with the log10‐transformed mean bird densities per km^2^ as outcome variable. The fixed factors entered into the full model included farm type (H, R‐W and R‐NW, with R‐NW as reference class), month (September, October, December, January, March and April, with the first month of the available bird counts, September, as reference), distance from farm (buffer rings 0–1, 1–3, 3–6 or 6–10 km, with the immediate surroundings of the poultry house at 0–1 km as reference) and the interaction terms farm type × month, farm type × distance and distance × month. Model selection was based on Akaike's Information Criterion (AIC), with the lowest AIC indicating the best fit (Burnham & Anderson, 2010).

First, the random part of the model (intercept and slope) was tested in the full models based on the restricted maximum‐likelihood (REML) method (Harville, 1977). The models with a random intercept for the farm location, and a random intercept for distance within the farm location, showed the best fit for each of the bird groups. Subsequent maximum‐likelihood (ML) estimation with the full models including these random effects showed that an autoregressive correlation structure of order 1 (AR1), to correct for correlations of observations between distances within the same farm location, improved model fit for all bird groups. Also, inclusion of a constant variance error function (varIdent) (Pinheiro et al., 2019), to take into account the heterogeneity in variances between farm types, resulted in the lowest AIC for all bird groups.

Next, to select the relevant fixed factors for the final models for each of the bird groups, we used a stepwise backward approach with single‐term deletions. All of these models were fitted with the previously determined random effects and AR(1) correlation structure, varIdent variance function and ML method.

To allow for comparisons of densities of birds around R‐NW, R‐W and H farms for the different months, the final model was reformulated by nesting farm type within month (Nelder, 1994). Geometric mean ratios (MR) were computed by applying an anti‐log transformation of the linear regression model coefficients, and predicted values were generated for the mean geometric bird densities for the different months and distances, within each of the different farm types.

Model assumptions were evaluated by QQ‐plots and a scatter plot of the residuals versus the predicted values and the fixed factors, respectively. Confidence intervals around the MR estimates were compared to evaluate differences between farm types, months and distances.

## 3 RESULTS

**FIGURE 2:**
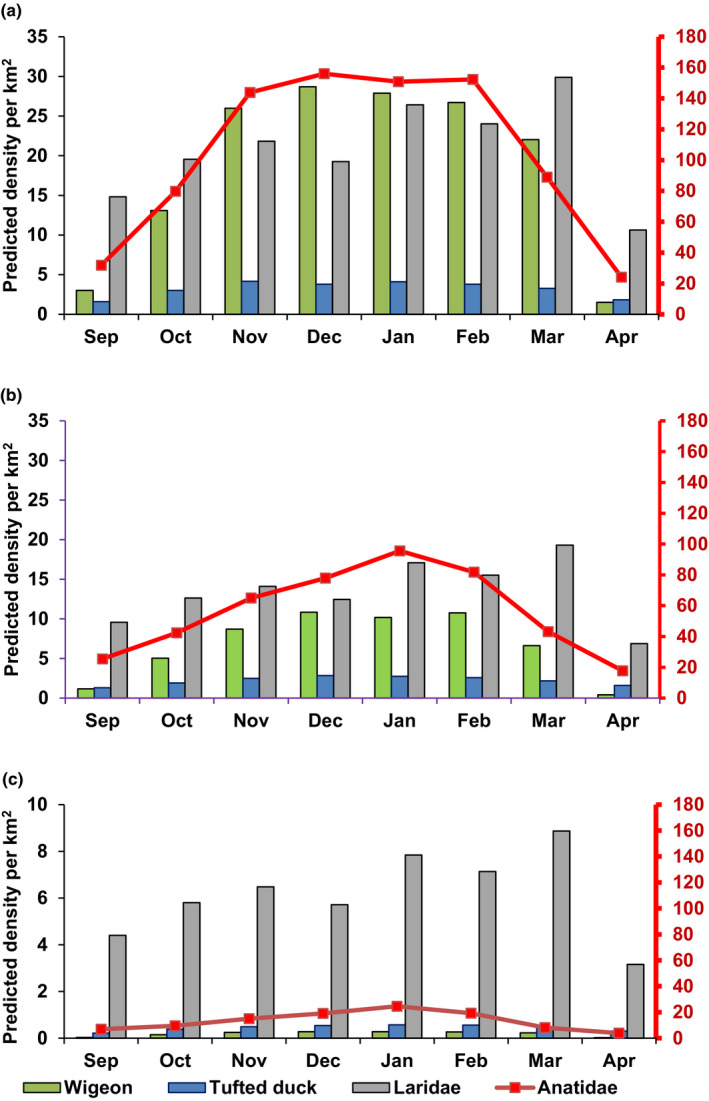
Model predictions of bird density per km^2^ between September and April for Eurasian wigeon (green), tufted ducks (blue), *Anatidae* (red*,* with density per km^2^ on secondary axis) and *Laridae* (grey) around (a) hotspot locations (H), (b) reference farms in a water‐rich area (R‐W), and (c) reference farms in a non‐water‐rich area (R‐NW). Note the differences in scaling on the Y‐axis for Figure 2c due to the much lower bird density per km^2^ in R‐NW compared to H and R‐W farms

For all farm types, that is for H, R‐W and R‐NW farms, predicted bird densities were higher between October and March (Eurasian wigeons, *Anatidae* and *Laridae*) and October and April (tufted ducks) compared to September (Table 1, Figure 2). The highest densities for the waterfowl group *Anatidae* were found between November and February, and for *Laridae,* maximum densities were not found until March (Figure 2a–c). The densities of tufted ducks were highest around H farms in November and of Eurasian wigeons in December (Figure 2a).

**TABLE 1:** Final model and model outputs for Eurasian Wigeon (A), *Anatidae* (B), tufted ducks (C) and *Laridae* (D). The model outputs represent mean ratio (MR) and 95% confidence intervals (CI) of the geometric mean bird densities/km^2^ obtained after anti‐log transformation of the linear regression model coefficients

A. Model estimates based on final model for Eurasian Wigeon with factors: Farm type + month + distance + farm type x month			B. Model estimates based on final model for *Anatidae* with factors: Farm type + month + distance + farm type x month		
Factor	MR	95% CI	Factor	MR	95% CI
*Intercept = September, distance 0–1 km. R‐NW farms = 0.01 (0–0.04)*			*Intercept = September, distance 0–1 km, R‐NW farms = 4.01 (2.32–6.92)*		
Oct^a^	4.32	3.77‐4.96	Oct^a^	1.37	1.26‐1.49
Nov	7.12	5.96‐8.52	Nov	2.16	1.95‐2.39
Dec	7.82	6.39‐9.58	Dec	2.76	2.48‐3.08
Jan	7.83	6.30‐9.74	Jan	3.55	3.18‐3.97
Feb	7.67	6.10‐9.63	Feb	2.77	2.48‐3.10
Mar	6.57	5.20‐8.31	Mar	1.18	1.06‐1.33
April	0.56	0.44‐0.71	April	0.55	0.49‐0.62
Distance 1–3 km^b^	1.98	1.03‐3.82	Distance 1–3 km^b^	1.42	1.04‐1.93
Distance 3–6 km	3.35	1.74‐6.46	Distance 3–6 km	2.07	1.51‐2.82
Distance 6–10 km	4.44	2.30‐8.56	Distance 6–10 km	2.43	1.78‐3.32
Sept: R‐W farms^c^	33.03	6.13‐177.90	Sept: R‐W farms^c^	3.68	1.68‐8.08
Oct: R‐W farms	33.40	6.20‐179.88	Oct: R‐W farms	4.45	2.03‐9.77
Nov: R‐W farms	35.03	6.50‐188.67	Nov: R‐W farms	4.35	1.98‐9.55
Dec: R‐W farms	39.78	7.39‐214.24	Dec: R‐W farms	4.07	1.85‐8.94
Jan: R‐W farms	37.34	6.93‐201.12	Jan: R‐W farms	3.89	1.77‐8.53
Feb: R‐W farms	40.23	7.47‐216.67	Feb: R‐W farms	4.25	1.94‐9.34
Mar: R‐W farms	28.92	5.37‐155.75	Mar: R‐W farms	5.26	2.40‐11.56
April: R‐W farms	21.24	3.94‐114.42	April: R‐W farms	4.63	2.11‐10.17
Sept: H farms^c^	86.36	14.17‐526.34	Sept: H farms^c^	4.60	1.97‐10.74
Oct: H farms	86.82	14.24‐529.16	Oct: H farms	8.38	3.59‐19.57
Nov: H farms	104.68	17.17‐638.00	Nov: H farms	9.63	4.12‐22.49
Dec: H farms	105.26	17.27‐641.53	Dec: H farms	8.16	3.49‐19.06
Jan: H farms	102.24	16.77‐623.14	Jan: H farms	6.13	2.63‐14.33
Feb: H farms	99.95	16.40‐609.21	Feb: H farms	7.94	3.4‐18.53
Mar: H farms	96.23	15.79‐586.50	Mar: H farms	10.84	4.64‐25.33
April: H farms	78.16	12.82‐476.37	April: H farms	6.29	2.69‐14.70

C. Model estimates based on final model for Tufted ducks with factors: Farm type + month + distance + farm type x month + farm type x distance					
Factor	MR	95% CI	Factor	MR	95% CI
*Intercept = September, distance 0–1 km = 0.16 (0.08–0.35)*					
Oct^a^	1.74	1.64‐1.84			
Nov	2.23	2.07‐2.39			
Dec	2.45	2.27‐2.65			
Jan	2.57	2.37‐2.78			
Feb	2.52	2.32‐2.73			
Mar	2.20	2.03‐2.39			
April	1.38	1.27‐1.50			
Sept: R‐W farms^c^	1.64	0.50‐5.37	Sept: H farms^c^	3.09	0.86‐11.16
Oct: R‐W farms	1.38	0.42‐4.51	Oct: H farms	3.36	0.93‐12.11
Nov: R‐W farms	1.40	0.43‐4.56	Nov: H farms	3.60	1.00‐12.99
Dec: R‐W farms	1.44	0.44‐4.72	Dec: H farms	2.98	0.83‐10.73
Jan: R‐W farms	1.33	0.41‐4.35	Jan: H farms	3.09	0.86‐11.14
Feb: R‐W farms	1.28	0.39‐4.17	Feb: H farms	2.91	0.81‐10.49
Mar: R‐W farms	1.24	0.38‐4.04	Mar: H farms	2.87	0.79‐10.33
April: R‐W farms	1.44	0.44‐4.69	April: H farms	2.57	0.71‐9.25
Distance 1–3 km^d^	0.94	0.49‐1.81			
Distance 3–6 km	1.43	0.74‐2.75			
Distance 6–10 km	2.05	1.07‐3.95			
Distance 1–3 km: R‐W farms^e^	4.98	1.82‐13.59	Distance 1–3 km: H farms^e^	1.42	0.48‐4.24
Distance 3–6 km: R‐W farms	4.97	1.82‐13.58	Distance 3–6 km: H farms	3.55	1.19‐10.58
Distance 6–10 km: R‐W farms	3.38	1.24‐9.24	Distance 6–10 km: H farms	2.59	0.87‐7.72

D. Model estimates based on final model for *Laridae* with factors: Farm type + month		
Factor	MR	95% CI
*Intercept = September, R‐NW farms = 4.40 (2.78–6.96)*		
R‐W farms^f^	2.18	1.04‐4.54
H farms	3.37	1.53‐7.43
Oct^g^	1.32	1.26‐1.39
Nov	1.47	1.39‐1.56
Dec	1.30	1.22‐1.38
Jan	1.78	1.68‐1.90
Feb	1.62	1.52‐1.73
Mar	2.02	1.89‐2.15
April	0.72	0.67‐0.77

Note

The footnote symbols are given behind the first item of each factor (specific month, distance, etc.), but relate to all items of that factor.

H, hotspot farms infected with H5N8; R‐NW, reference farms in non‐water‐rich area; R‐W, reference farms in water‐rich area; Sept, September; Oct, October; etc.

^a^ Ratio of geometric mean bird density (BD) of specific month for R‐NW to the geometric mean BD in reference month Sept for R‐NW farms.

^b^ Ratio of geometric mean BD of specific distance to the geometric mean BD for reference distance 0‐1 km (for all farm types and months).

^c^ Ratio of geometric mean BD of specific month for R‐W or H‐W farms to the geometric mean BD of the same month for R‐NW farms.

^d^ Ratio of geometric mean bird density of specific distance for R‐NW farms to the geometric mean bird density for reference distance 0‐1 km for R‐NW farms.

^e^ Ratio of geometric mean BD of specific distance for R‐W or H‐W farms to the geometric mean BD of the same distance for R‐NW farms.

^f^ Ratio of geometric mean BD of R‐W or H farms to the geometric mean BD for R‐NW farms.

^g^ Ratio of geometric mean BD of specific month to the geometric mean BD in reference month Sept.

The best‐fitting linear models for Eurasian wigeons, *Anatidae* and tufted ducks included the interaction term between farm type and month (Table 1A–C). This indicates that, although for all farm types bird densities were higher during the fall and winter months for these waterfowl groups, the magnitude of the increase in mean densities was different between farm types. Generally, higher predicted densities were found around H and R‐W farms compared to R‐NW farms (Figure 2a–c).

We also observed differences in the magnitude of the effects of farm type on bird density levels between the bird groups. Depending on the month, geometric mean densities ratios (MR, Table 1A) showed 21 (95% CI: 4–114 in April) to 40 (7–217 in February) times higher densities of Eurasian wigeons around R‐W compared to R‐NW farms. For H farms, the differences compared to R‐NW farms were even larger, ranging from 78 (13–476 in April) to 105 (17–642 in December) times higher densities. For *Anatidae,* bird densities between October and April were between 4 (95% CI: 2–8) and 11 (5–25) times higher for R‐W or H farms compared to R‐NW farms (Table 1B). For tufted ducks, densities around R‐W and H farms were up to 4 times higher, but the 95% CI around the MR in most cases included 1, suggesting that mean densities for R‐W and H farms were not significantly different from R‐NW farms (Table 1C). The final model for *Laridae* did not include an interaction term for farm type and month, but based on the MR for farm type alone, R‐W and H farms had 2 (95% CI: 1–5) to 3 (2–7) times higher densities compared to R‐NW farms (Table 1D).

**FIGURE 3:**
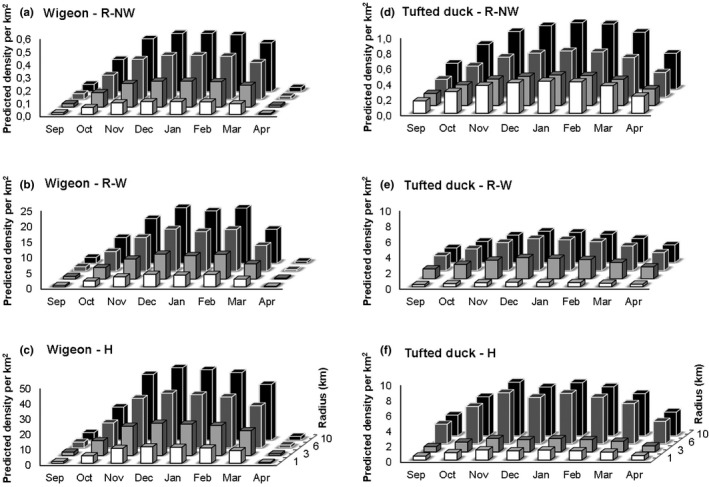
Predicted density of birds per km^2^ for the 0–1 (white), 1–3 (light grey, black border), 3–6 (darker grey, white border) and 6–10 km rings around the farms (black) for (a) Eurasian wigeon for reference farms in non‐water‐rich area (R‐NW), (b) water‐rich area (R‐W) and (c) around hotspot locations (H) and (d) for tufted ducks for R‐NW, (e) R‐W and (f) H farms. Note differences in scale on Y‐axis due to large differences in bird densities

Bird densities in the buffer rings at 1–3, 3–6 and 6–10 km distance from the farm were generally higher for all farm types for Eurasian wigeons (Table 1A; Figure 3a–c) and *Anatidae* (Table 1B) at increasing distances from the farm. Such a clear trend of higher densities at increasing distances was not found for *Laridae* nor for tufted ducks. For *Laridae,* distance was not a relevant factor in the model (Table 1D). The best‐fitting model for tufted ducks included the interaction term between farm type and distance, indicating that distance effects were different per farm type (Table 1C), which is also visualized in Figure 3. Especially for R‐NW farms, the higher densities were most clear from buffer ring 3–6 km onwards (Figure 3d), but for R‐W (Figure 3e) and H farms (Figure 3f), higher densities were also found at shorter distances from the farm (Table 1C; Figure 3).

## 4 DISCUSSION

This study has shown that a marked increase in HPAI high‐risk birds around poultry farms occurs from October to April. This increase was much more pronounced for farms in wetlands compared to farms not located in wetlands. Also, large differences between bird groups with regard to the magnitude of the increase in density were identified. The most striking increase was found for the Eurasian wigeons, with several tenfolds higher bird densities compared to farms not located in wetland areas. Eurasian wigeons were one of the predominant species with massive mortality in 2016–2017 and were also found in the vicinity of poultry farms with phylogenetically related H5N8 virus (Beerens et al., 2017).

Within the wetland areas, a clear difference in bird densities between H and R‐W farms could not be shown. This may be due to the small number of outbreak farms and reference farms in wetland areas combined with the large variations in bird densities between farms, limiting the power of the study. Another explanation may be that bird densities were similarly high around outbreak and reference farms in wetland areas, but that other unknown environmental or farm‐related factors, such as differences in the level of biosecurity, may have played a role.

Densities around H farms for Eurasian wigeons and *Anatidae*, that is waterfowl species, and not gulls, were significantly higher for the different buffer rings compared to the 0–1 km buffer ring, showing an almost linear trend of higher densities at increasing distances. This is not surprising, as a large part of the 0–1 km buffer ring around the farm is likely to consist of the farm yard, which included farm buildings and paved roads, which is an unattractive habitat for waterfowl, but not so unattractive for gulls. For tufted ducks, also waterfowl of the *Anatidae* family, such a linear trend was not seen. Tufted ducks are found on large open waterbodies such as lakes, and rarely fly over land between foraging and roosting sites (Kleyheeg et al., 2017). Consequently, it is likely that the tufted duck densities were strongly influenced by the coincidental presence of a large lake near the farms. A large lake is more likely to be nearby H and R‐W farms in the wetland areas than nearby R‐NW farms, but unlikely to be within the 0–1 and 1–3 km buffer rings for all farm types, which is consistent with our findings. Although it is unlikely to change the main conclusions of this study, it should be noted that bird densities in non‐wetland areas were generally based on a less fine‐meshed counting area grid with lower counting coverage compared to wetlands. This may have affected accuracy of estimated densities around R‐NW farms to different extents between birds that require a specific localized habitat, such as tufted ducks, and species like gulls and many terrestrial *Anatidae* species.

Although it was shown that high densities of the studied bird groups were present around farms, this study does not provide data on how many of these birds were infected, and to what extent their presence contributed to disseminating virus in the farms’ surroundings. Hence, we cannot draw conclusions on exact roles of specific wild bird species in the epidemiological processes at the wildlife/domestic interface, nor on that of other birds not included in this study. Nevertheless, the combined data of our study and published reports do suggest involvement of these bird groups in the HPAI outbreaks. Beerens et al. (2017) showed that the HPAIV from H farms was genetically closely related to virus detected in tufted ducks, greylag geese (*Anser anser*), mallards and Eurasian wigeons found dead within 3–20 km of the farms and that for the seven outbreaks used in this study, lateral spread between farms could be ruled out. The timing of events provides further support for wild bird to poultry transmission, rather than spill over from poultry to the wild birds around the farms (Alarcon et al., 2018). In fact, an important outcome of this study is that the timing of peak densities around the farms coincided with the timing of outbreaks in poultry, between late November and late December 2016 and also with most wild bird cases between half November and half December (OIE, 2017). The first massive wave of wild bird die‐offs started early November and consisted mainly of tufted ducks, with the majority (95%) of tufted duck cases being reported to OIE between 8 and 20 November. This was followed by the first reported deaths in Eurasian wigeons from 21 November onwards, with most (92%) of Eurasian wigeon cases reported between 5 and 18 December (OIE, 2017). This temporal pattern corresponds with the peak densities around H farms for tufted ducks in November and for Eurasian wigeons in December according to our analyses. Also from late November onwards, until March, deaths in *Laridae* species and other scavengers such as raptors and corvids were increasingly reported, but in much smaller numbers and without a clear pattern in time (Kleyheeg et al., 2017; Poen et al., 2018; OIE, 2017). Backyard poultry were of limited epidemiological relevance, with 10 reported outbreaks between 10 November 2016 and 22 March 2017 (OIE, 2017).

In contrast with the H5N6 affecting poultry and wild birds in 2017–2018 that evolved locally in wild birds from persisting H5N8 2016–2017 viruses and reassortment with wild host reservoir LPAI viruses (Alarcon et al., 2018; Beerens et al., 2018; Poen et al., 2019), H5N8 2016–2017 originated from poultry in South‐East Asia and was spread to Europe via migration of waterfowl with subsequent diffusion in wild birds (Lee et al., 2015, 2017; Mine et al., 2019). Between bird groups in this study, but also within the *Anatidae* family, the different species can have played different roles in the wildlife/domestic interface. Tufted ducks and wigeons are both known as long‐distance migratory birds, but which species introduced the virus through long‐distance migration has remained unknown to this date. Moreover, which (migratory or resident) species acted like spreaders, maintenance or bridge hosts or were merely dead‐end hosts that quickly died upon exposure to other infected birds, cannot be determined. An increase in antibody incidence in mallards was found at the end of the 2016–2017 outbreaks, corresponding with a shift from mortality in tufted ducks and wigeons to mallards later in the outbreak, suggesting that mallards might be more resistant to disease and might act as reservoir species (Poen et al., 2018). Dabbling ducks, like tufted ducks, generally show higher AIV prevalence, which may be related to higher risks of waterborne infection due to their foraging behaviour, but also due to intrinsic differences in receptivity to AIV infection between species (Gaidet et al., 2012). But even though prevalence of infection was high in tufted ducks, they generally live at a considerable distance from farms. Hence, other birds, including non‐aquatic species, may have acted as local spreaders or bridge species between HPAI high‐risk birds and domestic poultry (Caron et al., 2014), including those not belonging to the *Anatidae* and *Laridae* families included in this study. Also, exact mechanisms and relative roles of animal and human vectors and fomites in transmission of HPAIV from wild birds outside, to poultry inside the farms, including rodents (Velkers, Blokhuis, Veldhuis Kroeze, & Burt, 2017), are still poorly understood.

This work has contributed with some valuable insights into the spatio‐temporal density dynamics of a selection of HPAI high‐risk birds in the Netherlands. Especially where waterbodies and foraging land are nearby, species, for example the Eurasian wigeon, can reach densities tenfolds higher than elsewhere, whereas tufted ducks are numerous where large open waterbodies are nearby. This study also highlights large gaps in knowledge needed to facilitate identifying priority areas for surveillance and preventive measures (EFSA AHAW Panel, 2017). For instance, for estimating the relative contribution of potential factors to the risk of infection, for example the presence or density of certain species, relative distance from the farm and different environmental or ecological drivers, more detailed analyses as for instance done by (Gaidet et al., 2012; Galletti et al., 2018; Si et al., 2013) are needed. This would require data on more (infected) farms, by compiling data over multiple years, countries and perhaps LPAIV infections as well. Also, studies combining all available epidemiological, ecological and genetic data, for example such as done by Mulatti et al. (2018) for the HPAI H5N8 outbreaks in Northern Italy, can contribute to current knowledge. Moreover, mathematical models to study the complex dynamics of host–pathogen interactions in a multi‐host system can help identify transmission mechanisms, enabling prediction and possibly prevention of outbreaks (Caron, De Garine‐Wichatitsky, Ndlovu, & Cumming, 2012; Lisovski et al., 2018). Such approaches have for instance revealed the potential relevance of understudied bridge hosts (Caron et al., 2014) and identified a more broad host range of species to consider as maintenance hosts (Caron, Cappelle, & Gaidet, 2017; Lisovski et al., 2018). Lisovski et al. (2018) revealed a key mechanism in virus amplification, that is the constant flow and replacement of migratory birds during peak migration, and stressed the need to collect host demographical parameters, such as population density, timing of birth and turnover of migrants in surveillance studies. Future active and passive surveillance efforts would therefore highly benefit from data collection informed by such eco‐epidemiological models, and should include different actors at the wildlife/domestic interface, including long‐ and short distance spreaders, maintenance or bridge hosts (Alarcon et al., 2018; Caron et al., 2017).

In conclusion, wild birds can pose a substantial risk for primary introduction of AIV for commercial poultry, but means to influence their presence or infection status are limited. Consequently, the main focus should be on surveillance, biosecurity measures and decisions on establishments of new poultry farms, to reduce risk of HPAI outbreaks, using a risk‐based targeted approach, based on knowledge of environmental, ecological and epidemiological drivers for wild bird presence and abundance, taking the complex multi‐host dynamics into account.

## CONFLICT OF INTEREST

The authors declare no conflict of interest.

## Supporting information

Fig S1

Table S1
