# Supplementary figures and images for: Association of wild bird densities around poultry farms with the risk of highly pathogenic avian influenza virus subtype H5N8 outbreaks in the Netherlands, 2016
Source: Transbound Emerg Dis. 2020 May 18;68(1):76–87. doi: 10.1111/tbed.13595 (PMC8048466; doi:10.1111/tbed.13595)

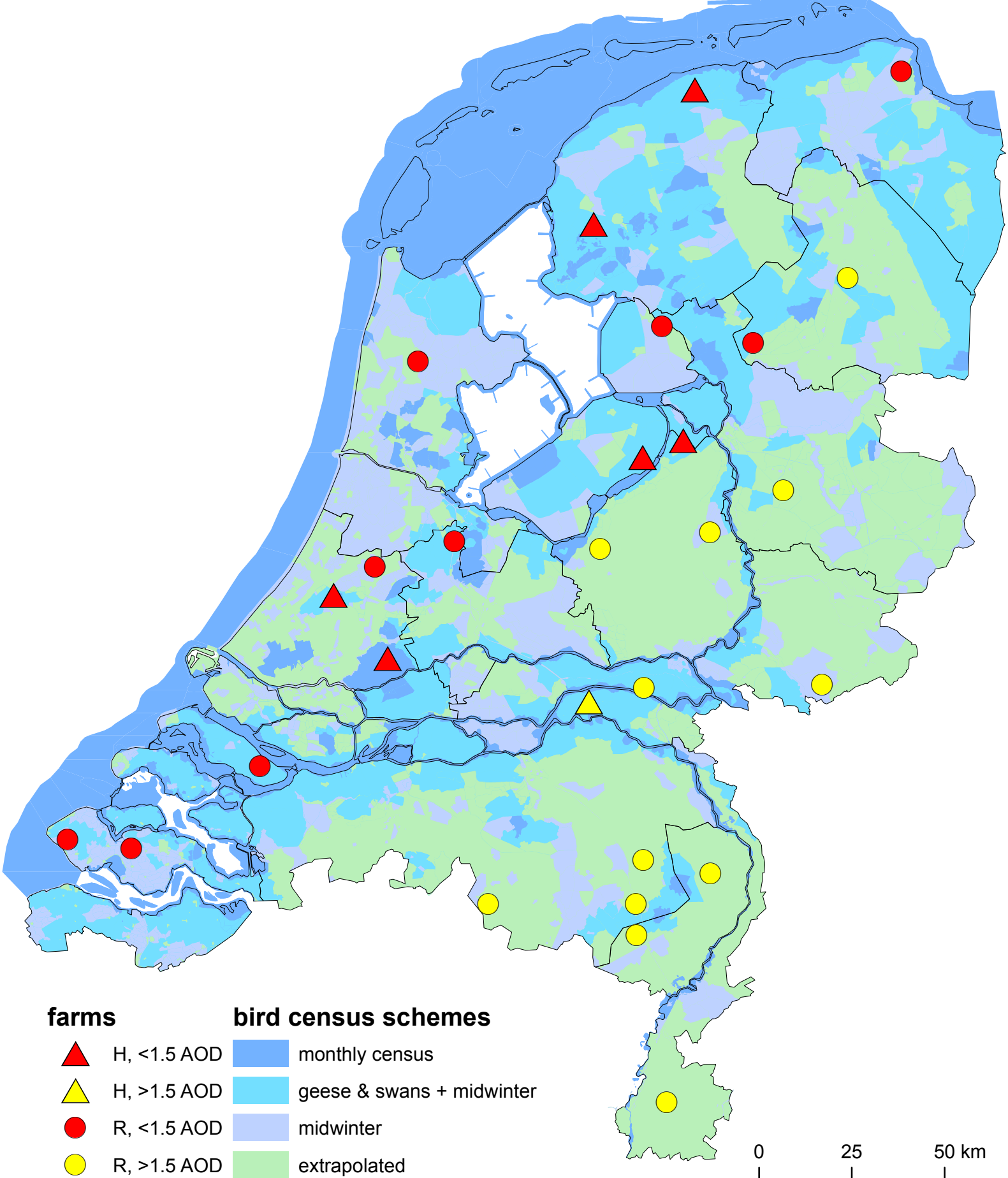

SUPPORTING INFORMATION FIGURE S1

Supplement: Supplementary file 1 — Fig S1 [file TBED-68-76-s002.pdf]
