# Supplementary material for: Association of wild bird densities around poultry farms with the risk of highly pathogenic avian influenza virus subtype H5N8 outbreaks in the Netherlands, 2016
Source: Transbound Emerg Dis. 2020 May 18;68(1):76–87. doi: 10.1111/tbed.13595 (PMC8048466; doi:10.1111/tbed.13595)
Supplement: Supplementary file 2 — Table S1 [file TBED-68-76-s001.docx]

**Supporting Information**

Association of wild bird densities around poultry farms with the risk of highly pathogenic avian influenza virus subtype H5N8 outbreaks in the Netherlands, 2016

Francisca C. Velkers^1^*, Thijs T.M. Manders^1^, Johannes C.M. Vernooij^1^, Julia Stahl^2^, Roy Slaterus^2^, J. Arjan Stegeman^1^

TABLE S1. Locations, poultry types and Amsterdam Ordnance Datum (AOD) level for outbreak and reference farms throughout the Netherlands.

| **Outbreak farms (H)** | | |  | **Reference farms (R)** | | | |
| --- | --- | --- | --- | --- | --- | --- | --- |
| Town | Coordinates† | Poultry type | AOD | Town | Coordinates† | Poultry type | AOD |
| Biddinghuizen | 52.4561429, 5.7590786 | Fattening ducks | -2.1§ | Den Bommel | 51.7025734, 4.2605799 | Layer (with outdoor range) ‡ | 1.4§ |
| Abbega | 53.0222643, 5.5671907 | Layer | 0.6§ | Loenen aan de Vecht | 52.2522851, 5.0145300 | Broiler breeder | 1.4§ |
| Kamperveen | 53.1775989, 6.2624749 | Fattening ducks | -0.1§ | Meliskerke | 52.3556308, 5.6769450 | Rearing hens | 0.7§ |
| Hiaure | 53.0995009, 6.0341250 | Layer (with outdoor range)† | 0.7§ | Lewedorp | 51.4970046, 3.7661538 | Layer | 1.5§ |
| Boven-Leeuwen | 51.8641660, 5.5443745 | Broiler breeder | 4.6 | Godlinze | 53.3923555, 6.8700395 | Layer | 1.4§ |
| Stolwijk | 51.9677566, 4.7555454 | Pet shop wholesale | -1.2§ | Havelte | 52.7314706, 6.2030296 | Layer, organic (outdoor range) ‡ | 1.3§ |
| Zoeterwoude | 52.1195011, 4.5397239 | Layer | -1.8§ | Luttelgeest | 52.7736061, 5.8393609 | Layer | -1.8§ |
|  |  |  |  | Heerhugowaard | 52.2209455, 5.6127602 | Layer | -1.9§ |
|  |  |  |  | Ter Aar | 52.1894245, 4.7010603 | Layer | 0.1§ |
|  |  |  |  | Putten | 52.2352592, 5.5901718 | Layer, cage | 10.6 |
|  |  |  |  | Reusel | 51.3727487, 5.1524765 | Layer | 31.7 |
|  |  |  |  | Someren | 51.3742318, 5.7242187 | Broiler breeder | 27.1 |
|  |  |  |  | Deurne | 51.4796980, 5.7536464 | Fattening ducks | 22.3 |
|  |  |  |  | Meterik | 51.4455032, 6.0137332 | Layer | 27.2 |
|  |  |  |  | Nederweert | 51.2975431, 5.7260335 | Layer | 31.5 |
|  |  |  |  | Breeden-broek | 52.3083800, 4.8481890 | Broiler breeder rearing | 17.8 |
|  |  |  |  | Beemte Broekland | 52.2735941, 6.0243731 | Layer breeder | 4.8 |
|  |  |  |  | Marienheem | 52.3732889, 6.3162312 | Broiler breeder rearing | 7.8 |
|  |  |  |  | Zwiggelte | 52.8406787, 6.7216819 | Meat turkeys | 16.4 |
|  |  |  |  | Herveld | 51.4255980, 5.4145116 | Broiler Grandparent | 9.9 |
|  |  |  |  | Hulsberg | 51.2243471, 4.4227164 | Layer | 133.7 |
| AOD, Amsterdam Ordnance Datum (reference for the European Vertical Reference System), a geodetic reference frame for land height and was determined using Geoweb NAPinfo (Rijkswaterstaat, the Netherlands ).  †Latitude and longitude coordinates of the location of the poultry house.  ‡ Note that from November 2016– April 2017 all free range poultry was housed indoors.  § Farms with AOD level below 1.5, with reference level 0 corresponding approximately to sea level, were considered to be located in a water-rich environment and hence attractive for waterfowl. | | | | | | | |

TABLE S2. List of bird species analyzed for the categories *Anatidae* and *Laridae.*

| **Species** | **Category** | **Species** | **Category** | |
| --- | --- | --- | --- | --- |
| Barnacle goose (*Branta leucopsis*) | *Anatidae* | Tufted duck (*Aythya fuligula*) † | *Anatidae* | |
| Bewick‘s swan (*Cygnus columbianus bewickii*) | *Anatidae* | Tundra bean goose (*Anser serrirostris*) | *Anatidae* | |
| Brant goose (*Branta bernicla*) | *Anatidae* | Whooper swan (*Cygnus cygnus*) | *Anatidae* | |
| Canada goose (*Branta canadensis*) | *Anatidae* |  |  | |
| Common goldeneye (*Bucephala clangula*) | *Anatidae* | Black-headed gull (*Chroicocephalus ridibundus*) | *Laridae* | |
| Common merganser (*Mergus merganser*) | *Anatidae* | European herring gull (*Larus argentatus*) | *Laridae* | |
| Common pochard (*Aythya ferina*) | *Anatidae* | Greater black-backed gull (*Larus marinus*) | *Laridae* | |
| Common shelduck (*Tadorna tadorna*) | *Anatidae* | Mew gull (*Larus canus*) | *Laridae* | |
| Common teal (*Anas crecca*) | *Anatidae* |  |  | |
| Egyptian goose (*Alopochen aegyptiaca*) | *Anatidae* |  |  | |
| Eurasian wigeon (*Anas penelope*) † | *Anatidae* |  |  | |
| Gadwall (*Anas strepera*) | *Anatidae* |  |  | |
| Greater white-fronted goose (*Anser albifrons*) | *Anatidae* |  |  | |
| Greylag goose (*Anser anser*) | *Anatidae* |  |  | |
| Mallard (*Anas platyrhynchos*) | *Anatidae* |  |  | |
| Mute swan (*Cygnus olor*) | *Anatidae* |  |  | |
| Northern pintail (*Anas acuta*) | *Anatidae* |  |  | |
| Northern shoveler (*Anas clypeata*) | *Anatidae* |  |  | |
| Pink-footed goose (*Anser brachyrhynchus*) | *Anatidae* |  |  | |
| Red-crested pochard (*Netta rufina*) | *Anatidae* |  |  | |
| Smew (*Mergellus albellus*) | *Anatidae* |  |  | |
| †Densities of Eurasian wigeon and tufted ducks were included in the analyses for the category of *Anatidae* and were analyzed separately as well. | | | |  |
